# Assessment of Health Information About COVID-19 Prevention on the Internet: Infodemiological Study

**DOI:** 10.2196/18717

**Published:** 2020-04-01

**Authors:** Ignacio Hernández-García, Teresa Giménez-Júlvez

**Affiliations:** 1 Department of Preventive Medicine Lozano Blesa University Clinical Hospital of Zaragoza Zaragoza Spain; 2 Department of Preventive Medicine Miguel Servet University Hospital of Zaragoza Zaragoza Spain

**Keywords:** COVID-19, coronavirus, prevention, internet, information, evaluation, authorship, World Health Organization, official public health organizations, digital media, infodemic, infodemiology

## Abstract

**Background:**

The internet is a large source of health information and has the capacity to influence its users. However, the information found on the internet often lacks scientific rigor, as anyone may upload content. This factor is a cause of great concern to scientific societies, governments, and users.

**Objective:**

The objective of our study was to investigate the information about the prevention of coronavirus disease 2019 (COVID-19) on the internet.

**Methods:**

On February 29, 2020, we performed a Google search with the terms “Prevention coronavirus,” “Prevention COVID-19,” “Prevención coronavirus,” and “Prevención COVID-19”. A univariate analysis was performed to study the association between the type of authorship, country of publication, and recommendations to avoid COVID-19 according to the World Health Organization (WHO).

**Results:**

In total, 80 weblinks were reviewed. Most of them were produced in the United States and Spain (n=58, 73%) by digital media sources and official public health organizations (n=60, 75%). The most mentioned WHO preventive measure was “wash your hands frequently” (n=65, 81%). A less frequent recommendation was to “stay home if you feel unwell” (n=26, 33%). The analysis by type of author (official public health organizations versus digital media) revealed significant differences regarding the recommendation to wear a mask when you are healthy only if caring for a person with suspected COVID-19 (odds ratio [OR] 4.39). According to the country of publication (Spain versus the United States), significant differences were detected regarding some recommendations such as “wash your hands frequently” (OR 9.82), “cover your mouth and nose with your bent elbow or tissue when you cough or sneeze” (OR 4.59), or “stay home if you feel unwell” (OR 0.31).

**Conclusions:**

It is necessary to urge and promote the use of the websites of official public health organizations when seeking information on COVID-19 preventive measures on the internet. In this way, users will be able to obtain high-quality information more frequently, and such websites may improve their accessibility and positioning, given that search engines justify the positioning of links obtained in a search based on the frequency of access to them.

## Introduction

Internet access has increased worldwide during the past decade, reaching 79.6% of the European population and 48% of the world population in 2017 [1]. In the United States, 90% of adults access the internet [2] and 53.1% look for health information online [3].

As with previous epidemics such as Ebola or Zika infections, the internet has become a favored mechanism for the spread of misinformation [4,5]. This has implications for public health behavior and health-related decision making [6].

At present, an outbreak of coronavirus disease 2019 (COVID-19) has occurred and has spread throughout China and to dozens of countries [7]. As in other epidemics, people want to know what can be done to prevent and treat the disease [6]. Since there is currently no vaccine or specific antiviral treatment, the application of preventive measures is essential.

In this context, we aimed to conduct an infodemiological study [8,9] to investigate the information about the prevention of COVID-19 available on the internet.

## Methods

On February 29, 2020, we performed a Google search and selected the first 20 links [5] of the Google search results, excluding advertisements. The search terms used were “Prevention coronavirus,” “Prevention COVID-19,” “Prevención coronavirus,” and “Prevención COVID-19”. Two reviewers (HG-I and GJ-T) viewed the links independently, and the following information was extracted from each link: type of authorship (official public health organizations, scientific societies, digital media, libraries, private health care system, articles from biomedical journals, or other), language, country of publication, and recommendations to avoid COVID-19. The information was obtained by making up to four clicks on the different sublinks of each link, as has been done in other studies [10,11]. Subsequently, the degree of adherence to the following World Health Organization (WHO) basic protective measures against the new coronavirus in force on February 29, 2020, was checked: wash your hands frequently; maintain at least 1 meter (3 feet) distance between yourself and anyone who is coughing or sneezing; avoid touching eyes, nose, and mouth; cover your mouth and nose with your bent elbow or tissue when you cough or sneeze (then dispose of the used tissue immediately); stay home if you feel unwell; if you develop fever, cough, and difficulty breathing, seek medical advice promptly (call in advance and tell your provider of any recent travel); if you are healthy, you only need to wear a mask if you are taking care of a person with suspected COVID-19; and wear a mask if you are coughing or sneezing [12].

We performed a descriptive analysis of all the variables and evaluated the association of the independent variables (type of authorship and country of publication) with the degree of adherence to the WHO basic protective measures by means of a chi-square test or Fisher exact test. When a significant association was found (*P*<.05), this was quantified with the odds ratio (OR) and its 95% CI obtained from univariate logistic regression analysis. The agreement between the two reviewers regarding the adherence to the WHO basic protective measures was analyzed using the Kappa index. All analyses were performed using SPSS v20.0 (IBM Corp) and EpiInfo (Centers for Disease Control and Prevention).

## Results

In total, 80 weblinks were reviewed (Textbox 1). Most of them were produced in the United States and Spain (n=58, 73%) by digital media and official public health organizations (n=60, 75%; Table 1). There were no discrepancies between the authors regarding the degree of adherence to the WHO basic protective measures (Kappa=1).

In addition, information that was ambiguous or did not adhere to the WHO recommendations was found in 8 weblinks (5 from Spain and 3 from the United States; 6 of the 8 were from digital media). In particular, 3 Spanish links indicated “maintain a distance of approximately one meter between people.” One Spanish link mentioned that “for people without respiratory symptoms a surgical mask is not required, although masks can be worn in some countries according to local cultural customs.” One link in Spain and another in the United States specified that “someone should only wear a mask if a healthcare professional recommends it.” One link in the United States mentioned, “If you're going to around a lot of sick people, like if you're visiting a friend in the hospital, a mask might be a good idea,” and one link in the United States recommended, “Stay three feet away from people when you talk to them.”

**Electronic addresses of the 80 weblinks by search term.**

**Search term: Prevention coronavirus**

- https://www.cdc.gov/coronavirus/2019-ncov/about/prevention-treatment-sp.html
- https://www.cdc.gov/coronavirus/2019-ncov/about/prevention-treatment.html
- https://www.ecdc.europa.eu/en/current-risk-assessment-novel-coronavirus-situation
- https://www.who.int/emergencies/diseases/novel-coronavirus-2019/advice-for-public
- https://www.who.int/emergencies/diseases/novel-coronavirus-2019/technical-guidance/infection-prevention-and-control
- https://choice.npr.org/index.html?origin=https://www.npr.org/2020/02/27/810016611/coronavirus-101-what-you-need-to-know-to-prepare-and-prevent
- https://www.nytimes.com/2020/02/26/health/coronavirus-cdc-usa.html
- https://cuidateplus.marca.com/enfermedades/infecciosas/Coronavirus.html
- https://www.osha.gov/SLTC/covid-19/
- https://www.conehealth.com/services/primary-care/coronavirus-get-the-facts-on-symptoms-and-prevention-with-cynthi/
- https://edition.cnn.com/2020/02/28/health/how-to-wash-hands-coronavirus-trnd/index.html
- https://www.nbcnews.com/health/health-news/main-focus-preventing-coronavirus-spread-should-be-hand-hygiene-not-n1144346
- https://www.businessinsider.com/wuhan-coronavirus-face-masks-not-entirely-effective-2020-1?IR=T
- https://abc7news.com/5971803/
- https://www.mobihealthnews.com/news/coronavirus-prevention-may-be-your-pocket
- https://www.washingtonpost.com/gdpr-consent/?next_url=https%3a%2f%2fwww.washingtonpost.com%2fhealth%2f2020%2f02%2f26%2f how-to-prepare-for-coronavirus%2f
- https://www.cnbc.com/2020/02/26/cdc-confirms-first-possible-community-spread-coronavirus-case-in-us.html
- https://foreignpolicy.com/2020/02/28/taiwan-who-coronavirus-china-international-organizations/
- https://parade.com/987803/lisamulcahy/coronavirus/
- https://www.canada.ca/en/public-health/services/diseases/2019-novel-coronavirus-infection/prevention-risks.html

**Search term: Prevention COVID-19**

- http://www.euro.who.int/en/health-topics/health-emergencies/coronavirus-covid-19/news/news/2020/2/joint-who-and-ecdc-mission-in-italy-to-support-covid-19- control-and-prevention-efforts
- https://openwho.org/courses/COVID-19-IPC-EN
- https://www.ecdc.europa.eu/en/novel-coronavirus-china
- https://www.ecdc.europa.eu/en/publications-data/infographic-covid-19
- http://bvsalud.isciii.es/covid-19/
- https://abc7news.com/5971803/
- https://www.japantimes.co.jp/opinion/2020/02/27/editorials/covid-19-preventing-medical-system-breakdown/#.XlrmyahKg2w
- https://jamanetwork.com/journals/jama/fullarticle/2762130
- https://www.iata.org/contentassets/7e8b4f8a2ff24bd5a6edcf380c641201/airport-preventing-spread-of-coronavirus-disease-2019.pdf
- https://www.cdc.gov/coronavirus/2019-ncov/about/prevention-treatment-sp.html
- https://www.cdc.gov/coronavirus/2019-ncov/index-sp.html
- https://www.osha.gov/SLTC/covid-19/controlprevention.html
- https://www.cnbc.com/2020/02/27/coronavirus-latest-updates-outbreak.html
- https://www.mica.edu/campus-operating-status-updates/coronavirus/best-practices-and-preventive-measures/
- https://www.kuow.org/stories/new-coronavirus-cases-found-in-king-and-snohomish-counties
- https://www.euronews.com/2020/02/26/coronavirus-prevention-how-effective-are-masks-closed-borders-screenings-and-quarantines
- https://www.health.govt.nz/our-work/diseases-and-conditions/covid-19-novel-coronavirus/covid-19-novel-coronavirus-health-advice-general-public
- https://www.canada.ca/en/public-health/services/diseases/2019-novel-coronavirus-infection/prevention-risks.html
- https://www.bmj.com/content/368/bmj.m810
- https://vietnamnews.vn/society/652839/pm-pushes-for-covid-19-preventive-measures.html

**Search term: Prevención COVID-19**

- https://www.cdc.gov/coronavirus/2019-ncov/index-sp.html
- https://www.saludcastillayleon.es/profesionales/es/enfermedades-infecciosas/nuevo-coronavirus-covid-19/plan-especifico-prevencion-riesgos-laborales- nuevo-coronavi
- https://www.alimente.elconfidencial.com/bienestar/2020-02-29/coronavirus-covid19-que-es-sintomas-contagio_2431343/
- https://www.saludcastillayleon.es/profesionales/es/enfermedades-infecciosas/nuevo-coronavirus-covid-19
- https://www.who.int/es/emergencies/diseases/novel-coronavirus-2019/advice-for-public/q-a-coronaviruses
- https://www.ibsalut.es/es/info-ciudadania/cuidar-la-salud/3710-preguntas-y-respuestas-sobre-el-nuevo-coronavirus-2019-n-cov
- https://www.campusvirtualsp.org/es/curso/virus-respiratorios-emergentes-incluido-el-2019-ncov-metodos-de-deteccion-prevencion-respuesta
- https://www.alimente.elconfidencial.com/bienestar/2020-02-29/coronavirus-covid19-que-es-sintomas-contagio_2431343/
- https://www.semfyc.es/como-prevenir-infecciones-por-virus-respiratorios-como-el-coronavirus-que-causa-la-enfermedad-covid-19/
- http://bvsalud.isciii.es/covid-19/
- https://www.mscbs.gob.es/profesionales/saludPublica/ccayes/alertasActual/nCov-China/documentos/20200224.Preguntas_respuestas_COVID-19.pdf
- https://www.mscbs.gob.es/profesionales/saludPublica/ccayes/alertasActual/nCov-China/documentos/Documento_Control_Infeccion.pdf
- https://www.lasexta.com/noticias/internacional/coronavirus-covid19-que-puedes-hacer-protegerte-como-actuar_202002245e53fcca0cf2547d2a31e546.html
- https://www.unicef.org/es/historias/coronavirus-lo-que-los-padres-deben-saber
- https://www.lavanguardia.com/vida/20200229/473828128008/coronavirus-espana-madrid-barcelona-wuhan-china-italia-covid-19-contagios- sintomas-fallecidos-ultima-hora-hoy-en-directo.html
- https://www.univision.com/local/philadelphia-wuvp/prevencion-del-coronavirus-que-funciona-para-evitar-la-propagacion-de-covid-19
- http://bvsalud.isciii.es/covid-19/
- https://medlineplus.gov/spanish/ency/article/007768.htm
- https://sano-y-salvo.blogspot.com/2020/02/infografias-para-prevenir-la-infeccion.html
- https://www.bbc.com/mundo/noticias-51683330

**Search term: Prevención coronavirus**

- https://cuidateplus.marca.com/enfermedades/infecciosas/Coronavirus.html
- https://www.quironprevencion.com/es/campanas-prevencion-riesgos-laborales/coronavirus-covid-2019
- https://medlineplus.gov/spanish/coronavirusinfections.html
- https://www.cdc.gov/coronavirus/2019-ncov/about/prevention-treatment-sp.html
- https://www.saludcastillayleon.es/profesionales/es/enfermedades-infecciosas/nuevo-coronavirus-covid-19/plan-especifico-prevencion-riesgos-laborales- nuevo-coronavi
- https://elpais.com/elpais/2020/02/25/ciencia/1582645440_172885.html
- https://www.elperiodico.com/es/sanidad/20200225/coronavirus-que-es-sintomas-contagio-prevencion-7814261
- https://vacunasaep.org/profesionales/noticias/coronavirus-desarrollo-de-vacunas
- https://www.hola.com/estar-bien/20200123158838/coronavirus-sintomas-prevenir-contagio/
- https://www.alimente.elconfidencial.com/bienestar/2020-02-29/coronavirus-covid19-que-es-sintomas-contagio_2431343/
- https://www.diariocordoba.com/noticias/sociedad/que-es-coronavirus-sintomas-contagio-prevencion-virus_1351515.html
- https://www.who.int/csr/disease/coronavirus_infections/ipc-mers-cov/es/
- https://www.intramed.net/contenidover.asp?contenidoid=95410
- https://www.bbc.com/mundo/noticias-51683330
- https://www.semes.org/semes-divulgacion/medidas-de-prevencion-ante-la-neumonia-por-coronavirus/
- https://www.lavanguardia.com/seguros/empresa/20200217/473630100957/mwc-alerta-sanitaria-contagio-corona-virus-riesgos-laborales-seguros.html
- https://chile.gob.cl/chile/medidas-de-prevencion-ante-el-nuevo-coronavirus
- https://www2.cruzroja.es/-/-como-puedes-reducir-el-riesgo-de-infeccion-del-coronavirus-
- https://temas.sld.cu/coronavirus/coronavirus/medidas-preventivas/
- https://www.lavanguardia.com/ciencia/20200225/473756254816/coronavirus-covid-19-mascarilla-prevencion.html

**Table 1:** Characteristics of the 80 weblinks.

Characteristics		Frequency, n (%)
**Country of publication**		
	United States	30 (38)
	Spain	28 (35)
	Switzerland	6 (8)
	United Kingdom	3 (4)
	Sweden	3 (4)
	Canada	2 (3)
	Others	8 (10)
**Type of authorship**		
	Digital media	33 (41)
	Official public health organizations	27 (34)
	Libraries	6 (8)
	Scientific societies	3 (4)
	Articles from biomedical journals	2 (3)
	Private health care system	2 (3)
	Others	7 (9)
**Language**		
	Spanish	45 (56)
	English	35 (44)
**Available recommendation according to the World Health Organization**		
	Wash your hands frequently	65 (81)
	Maintain at least 1 meter distance	56 (70)
	Cover your mouth and nose when you cough or sneeze	54 (68)
	Avoid touching eyes, nose, and mouth	44 (55)
	Wear a mask if you are coughing or sneezing	39 (49)
	If you develop fever, cough, and difficulty breathing, seek medical advice	37 (46)
	If you are healthy, wear a mask if you are taking care of a person with suspected COVID-19^a^	37 (46)
	Stay home if you feel unwell	26 (33)

^a^COVID-19: coronavirus disease 2019.

Univariate analysis by type of author (official public health organizations versus digital media) revealed statistically significant differences regarding the recommendation to wear a mask if you are healthy only if caring for a person with suspected COVID-19 (OR 4.39; Table 2). The analysis according to country of publication (Spain versus the United States) detected statistically significant differences regarding some recommendations such as “wash your hands frequently” (OR 9.82), “cover your mouth and nose with your bent elbow or tissue when you cough or sneeze” (OR 4.59), or “stay home if you feel unwell” (OR=0.31; Table 3).

**Table 2:** Recommendations to avoid COVID-19 according to the World Health Organization and information about them available on the internet according to their authorship.

Recommendation, type of authorship		Available, n (%)	Unavailable, n (%)	Odds ratio (95% CI)	*P* value
**Wash your hands frequently (available n=65, unavailable n=15)**					
	Official public health organizations	23 (35)	4 (27)	2.16 (0.58-7.99)	.35
	Libraries	6 (9)	0 (0)	—^a^	.31
	Others	12 (19)	2 (13)	2.25 (0.42-12.09)	.46
	Digital media	24 (37)	9 (60)	1	—
**Cover your mouth and nose when you cough or sneeze (available n=54, unavailable n=26)**					
	Official public health organizations	19 (35)	8 (31)	1.98 (0.68-5.79)	.21
	Libraries	6 (11)	0 (0)	—	.07
	Others	11 (20)	3 (12)	3.06 (0.72-13.01)	.19
	Digital media	18 (33)	15 (58)	1	—
**Maintain at least 1 meter distance between yourself and anyone who is coughing or sneezing (available n=56, unavailable n=24)**					
	Official public health organizations	21 (38)	6 (25)	2.00 (0.63-6.33)	.24
	Libraries	6 (11)	0 (0)	—	.15
	Others	8 (14)	6 (25)	0.76 (0.21-2.72)	.68
	Digital media	21 (38)	12 (50)	1	—
**Avoid touching eyes, nose, and mouth (available n=44, unavailable n=36)**					
	Official public health organizations	16 (36)	11 (31)	2.24 (0.79-6.32)	.13
	Libraries	6 (14)	0 (0)	—	.008
	Others	9 (21)	5 (14)	2.77 (0.76-10.13)	.12
	Digital media	13 (30)	20 (56)	1	—
**If you develop fever, cough, and difficulty breathing, seek medical advice (call and tell your provider of any recent travel; available n=37, unavailable n=43)**					
	Official public health organizations	11 (30)	16 (37)	1.06 (0.38-2.99)	.92
	Libraries	5 (14)	1 (2)	7.69 (0.81-73.55)	.08
	Others	8 (22)	6 (14)	2.05 (0.58-7.29)	.27
	Digital media	13 (35)	20 (47)	1	—
**Stay home if you feel unwell (available n=26, unavailable n=54)**					
	Official public health organizations	12 (46)	15 (28)	2.13 (0.73-6.27)	.17
	Libraries	1 (4)	5 (9)	0.53 (0.06-5.21)	>.99
	Others	4 (15)	10 (19)	1.07 (0.72-4.28)	>.99
	Digital media	9 (35)	24 (44)	1	—
**Wear a mask if you are coughing or sneezing (available n=39, unavailable n=41)**					
	Official public health organizations	17 (44)	10 (24)	1.81 (0.64-5.09)	.27
	Libraries	1 (3)	5 (12)	0.21 (0.02-2.02)	.21
	Others	5 (13)	9 (22)	0.59 (0.16-2.14)	.43
	Digital media	16 (41)	17 (42)	1	—
**If you are healthy, wear a mask if you are taking care of a person with suspected COVID-19^b^ (available n=37, unavailable n=43)**					
	Official public health organizations	20 (54)	7 (16)	4.39 (1.45-13.32)	.008
	Libraries	1 (3)	5 (12)	0.31 (0.03-2.94)	.39
	Others	3 (8)	11 (26)	0.42 (0.09-1.79)	.32
	Digital media	13 (35)	20 (47)	1	—

^a^Not available.

^b^COVID-19: coronavirus disease 2019.

**Table 3:** Recommendations to avoid COVID-19 according to the World Health Organization and information about them available on the internet according to their country of publication.

Recommendation, type of authorship		Available, n (%)	Unavailable, n (%)	Odds ratio (95% CI)	*P* value
**Wash your hands frequently (available n=65, unavailable n=15)**					
	Spain	27 (42)	1 (7)	9.82 (1.14-84.61)	.03
	Switzerland	4 (6)	2 (13)	0.73 (0.11-4.77)	>.99
	Others	12 (19)	4 (27)	1.09 (0.27-4.39)	>.99
	United States	22 (34)	8 (53)	1	—^a^
**Cover your mouth and nose with your bent elbow or tissue when you cough or sneeze (available n=54, unavailable n=26)**					
	Spain	24 (44)	4 (15)	4.59 (1.27-16.53)	.02
	Switzerland	4 (7)	2 (8)	1.53 (0.24-9.68)	>.99
	Others	9 (17)	7 (27)	0.98 (0.29-3.34)	.98
	United States	17 (32)	13 (50)	1	—
**Maintain at least 1 meter distance between yourself and anyone who is coughing or sneezing (available n=56, unavailable n=24)**					
	Spain	22 (39)	6 (25)	1.57 (0.48-5.18)	.46
	Switzerland	4 (7)	2 (8)	0.86 (0.13-5.55)	>.99
	Others	9 (16)	7 (29)	0.55 (0.16-1.94)	.36
	United States	21 (38)	9 (38)	1	—
**Avoid touching eyes, nose, and mouth (available n=44, unavailable n=36)**					
	Spain	13 (30)	15 (42)	0.43 (0.15-1.25)	.12
	Switzerland	3 (7)	3 (8)	0.50 (0.09-2.94)	.65
	Others	8 (18)	8 (22)	0.50 (0.15-1.73)	.28
	United States	20 (46)	10 (28)	1	—
**If you develop fever, cough, and difficulty breathing, seek medical advice (call and tell your provider of any recent travel; available n=37, unavailable n=43)**					
	Spain	19 (51)	9 (21)	3.17 (1.08-9.31)	.04
	Switzerland	4 (11)	2 (5)	3.00 (0.47-19.04)	.37
	Others	2 (5)	14 (33)	0.21 (0.04-1.12)	.09
	United States	12 (32)	18 (42)	1	—
**Stay home if you feel unwell (available n=26, unavailable n=54)**					
	Spain	6 (23)	22 (41)	0.31 (0.09-0.99)	.045
	Switzerland	2 (8)	4 (7)	0.57 (0.09-3.61)	.67
	Others	4 (15)	12 (22)	0.38 (0.09-1.46)	.21
	United States	14 (54)	16 (30)	1	—
**Wear a mask if you are coughing or sneezing (available n=39, unavailable n=41)**					
	Spain	13 (33)	15 (37)	0.66 (0.24-1.87)	.44
	Switzerland	3 (8)	3 (7)	0.77 (0.13-4.43)	>.99
	Others	6 (15)	10 (24)	0.46 (0.13-1.59)	.22
	United States	17 (44)	13 (32)	1	—
**If you are healthy, wear a mask if you are taking care of a person with suspected COVID-19^b^ (available n=37, unavailable n=43)**					
	Spain	11 (30)	17 (40)	0.57 (0.19-1.61)	.29
	Switzerland	3 (8)	3 (7)	0.88 (0.15-5.05)	>.99
	Others	7 (19)	9 (21)	0.68 (0.20-2.31)	.54
	United States	16 (43)	14 (33)	1	—

^a^Not available.

^b^COVID-19: coronavirus disease 2019.

## Discussion

This study is the first to evaluate the adherence of the information available on the internet to the WHO basic protective measures against COVID-19. It shows a level of adherence that can be improved and a difficulty in obtaining such information, since it was only available in 32.5%-81.3% of the links.

The difficulty of finding WHO-promoted measures to prevent other infectious diseases on the internet has also been described previously by other authors, such as Covolo et al [13]. The authors, when studying the information on the internet about the pandemic flu vaccine, showed that only 80.3% (61/76) and 53.9% (41/76) of the websites they evaluated contained information on the indications and contraindications, respectively, of the vaccine that correctly adhered to the WHO guidelines [13].

Less than half of the weblinks provided information on the correct use of masks and, together with the fact that some of the links provided information that was ambiguous or did not adhere to the WHO guidance, may have contributed to the misuse of masks by the population and with the subsequent shortage of these devices that is occurring worldwide [14,15].

As with other studies that evaluated information on the internet on preventive measures for other infections [11], our work shows that, in general, official public health organizations provide more correct information on measures to avoid COVID-19, which confirms what other authors have said about the reliability of the information provided by such institutions [10,13]. However, the fact that only 34% (n=27/80) of the links referred to such organizations is an aspect that could be improved and shows the need to implement some interventions to increase the number of links of this type and their visibility on the internet. In addition, digital media must take responsibility for providing correct information and creating comprehension among citizens [16].

According to the analysis by country, the Spanish links provided more information on measures to prevent COVID-19 that adhered to the WHO than did the links produced in the United States. The measures to prevent COVID-19 by the Centers for Disease Control and Prevention [17] are the same as those of the WHO, and the proportion of links with information that was ambiguous or did not adhere to these guidelines is similar in terms of originating in the United States (n=3/30) and Spain (n=5/28). Therefore, an explanation for these differences could be that at the time of data collection, COVID-19 was considered to pose a moderate risk to public health in Spain (with 50 cases among 46 million people [18]), while in the United States, the problem was still far away (with 66 cases among 327 million people [18]). For this reason, the links from the United States did not provide as much information as the Spanish links on how to prevent COVID-19.

One of the limitations of our study is intrinsic to the nature of internet, namely that information changes continuously; like others [5,10,11,13,19], this paper analyzed the information available at a particular time. On the other hand, as in previous studies on other infectious diseases [5], only the first 20 links obtained were evaluated, because it has been observed that internet users only use the first two pages of results [20]. Likewise, the search was carried out only with the Google search engine because it is the most popular search engine, covering nearly 90% of the total online searches [21]. Finally, like other studies [11,13], the search terms were chosen by the authors assuming that an internet user would probably use one of them to perform simple searches on the web with respect to preventative measures for COVID-19.

In conclusion, it is necessary to urge and promote the use of the websites of official public health organizations (and specifically those originating from Spain for Spanish-speaking users) when seeking information on COVID-19 preventive measures on the internet. In this way, they will be able to obtain high-quality information more frequently, and such websites’ accessibility and positioning may improve, given that search engines justify the positioning of links obtained in a search based on the frequency of access to them.

## Abbreviations

COVID-19: coronavirus disease 2019
OR: odds ratio
WHO: World Health Organization

## References

[1] (2017). International Telecommunications Union/UNESCO.

[2] (2019). Pew Research Center.

[3] Din HN, McDaniels-Davidson C, Nodora J, Madanat H (2019). Profiles of a health information-seeking population and the current digital divide: cross-sectional analysis of the 2015-2016 California Health interview survey. J Med Internet Res.

[4] Oyeyemi SO, Gabarron E, Wynn R (2014). Ebola, Twitter, and misinformation: a dangerous combination?. BMJ.

[5] Venkatraman A, Mukhija D, Kumar N, Nagpal SJS (2016). Zika virus misinformation on the internet. Travel Med Infect Dis.

[6] Gesser-Edelsburg A, Diamant A, Hijazi R, Mesch GS (2018). Correcting misinformation by health organizations during measles outbreaks: a controlled experiment. PLoS One.

[7] Jernigan DB, CDC COVID-19 Response Team (2020). Update: public health response to the coronavirus disease 2019 outbreak - United States, February 24, 2020. MMWR Morb Mortal Wkly Rep.

[8] Eysenbach G (2002). Infodemiology: the epidemiology of (mis)information. Am J Med.

[9] Eysenbach G (2009). Infodemiology and infoveillance: framework for an emerging set of public health informatics methods to analyze search, communication and publication behavior on the Internet. J Med Internet Res.

[10] Betsch C, Wicker S (2012). E-health use, vaccination knowledge and perception of own risk: drivers of vaccination uptake in medical students. Vaccine.

[11] Hernández-García I, Giménez-Júlvez T (2018). [Assessment of health information available online regarding meningococcal B vaccine recommendations]. Rev Esp Salud Publica.

[12] World Health Organization.

[13] Covolo L, Mascaretti S, Caruana A, Orizio G, Caimi L, Gelatti U (2013). How has the flu virus infected the Web? 2010 influenza and vaccine information available on the Internet. BMC Public Health.

[14] (2020). World Health Organization.

[15] Shimizu K (2020). 2019-nCoV, fake news, and racism. Lancet.

[16] Leung CC, Lam TH, Cheng KK (2020). Mass masking in the COVID-19 epidemic: people need guidance. Lancet.

[17] (2019). Centers for Disease Control and Prevention.

[18] Departamento de Seguridad Nacional.

[19] Wiley KE, Steffens M, Berry N, Leask J (2017). An audit of the quality of online immunisation information available to Australian parents. BMC Public Health.

[20] McTavish J, Harris R, Wathen N (2011). Searching for health: the topography of the first page. Ethics Inf Technol.

[21] Kłak A, Gawińska E, Samoliński B, Raciborski F (2017). Dr Google as the source of health information – the results of pilot qualitative study. Polish Annals of Medicine.

